# An inter-order horizontal gene transfer event enables the catabolism of compatible solutes by *Colwellia psychrerythraea* 34H

**DOI:** 10.1007/s00792-013-0543-7

**Published:** 2013-05-15

**Authors:** R. Eric Collins, Jody W. Deming

**Affiliations:** 1 Department of Earth and Planetary Sciences, McGill University, Montréal, Canada; 2 Department of Natural Resource Sciences, McGill University, Montréal, Canada; 3 Present Address: Institute of Marine Science, University of Alaska Fairbanks, Fairbanks, USA; 4 School of Oceanography, University of Washington, Seattle, USA

**Keywords:** Compatible solutes, Halophiles, Psychrophiles, Genomics

## Abstract

*Colwellia* is a genus of mostly psychrophilic halophilic Gammaproteobacteria frequently isolated from polar marine sediments and sea ice. In exploring the capacity of *Colwellia psychrerythraea* 34H to survive and grow in the liquid brines of sea ice, we detected a duplicated 37 kbp genomic island in its genome based on the abnormally high G + C content. This island contains an operon encoding for heterotetrameric sarcosine oxidase and is located adjacent to several genes used in the serial demethylation of glycine betaine, a compatible solute commonly used for osmoregulation, to dimethylglycine, sarcosine, and glycine. Molecular clock inferences of important events in the adaptation of *C. psychrerythraea* 34H to compatible solute utilization reflect the geological evolution of the polar regions. Validating genomic predictions, *C. psychrerythraea* 34H was shown to grow on defined media containing either choline or glycine betaine, and on a medium with sarcosine as the sole organic source of carbon and nitrogen. Growth by 8 of 9 tested *Colwellia* species on a newly developed sarcosine-based defined medium suggested that the ability to catabolize glycine betaine (the catabolic precursor of sarcosine) is likely widespread in the genus *Colwellia*. This capacity likely provides a selective advantage to *Colwellia* species in cold, salty environments like sea ice, and may have contributed to the ability of *Colwellia* to invade these extreme niches.

## Introduction

Sea ice is an extreme environment characterized by the low temperature (−2 to –35 °C) and high salinity (35–270 %) of its brine inclusions, where the microbes reside (Junge et al. 2001; Collins et al. 2008). High salinity is known to induce several mechanisms for osmotolerance in Bacteria, Archaea, and Eukarya, with one of the most common being the intracellular accumulation of up to molar quantities of compatible solutes like glycine betaine (Bremer and Kramer 2000; Roberts 2005). These compounds may also be released into sea ice brine channels as organisms adjust to seasonal or diurnal changes in brine salinity or following viral lysis or cell death. The ability to transport and metabolize compatible solutes for growth would provide a selective advantage in such an environment. Horizontal gene transfer, an important component of microbial evolution, allows bacteria to rapidly adapt to new environments by importing large fragments of DNA from other microorganisms. Although difficult to identify in situ, recent investigations of sea ice as a hotspot for horizontal gene transfer have focused on its high concentrations of bacteria, viruses, and extracellular free DNA, among other contributing factors (Collins and Deming 2011a, b).

*Colwellia* is a genus of heterotrophic, halophilic marine Gammaproteobacteria containing 12 psychrophilic or psychrotolerant species (Deming et al. 1988), two of which are also barophilic. Isolates belonging to this genus have been cultured from sea ice, marine sediments, estuarine waters, and both epipelagic and hadalpelagic marine environments. The complete genome sequence of *Colwellia psychrerythraea* 34H, isolated from Arctic marine sediments (Huston et al. 2000), has been published (Methé et al. 2005). Prior work with *C. psychrerythraea* 34H describing cold-active enzymes (Huston et al. 2000, 2004), motility, amino acid incorporation, and increased production of EPS at subzero temperatures (Junge et al. 2003, 2006; Marx et al. 2009), and a psychrophilic phage-host system (Wells and Deming 2006a, b; Colangelo-Lillis and Deming 2013) is complemented by plentiful genomic evidence of adaptation to cold salty environments by this microorganism. In addition to genes involved in maintaining membrane fluidity at low temperature and the synthesis of extracellular polysaccharides and enzymes, the genome also encodes for the production, transport, and degradation of compatible solutes (Methé et al. 2005).

Compatible solutes are small, chemically inert neutral or zwitterionic molecules accumulated intracellularly by microorganisms to maintain turgor pressure and stabilize proteins in high osmolarity environments (Bremer and Kramer 2000; Roberts 2005). One of the most common compatible solutes is glycine betaine (*N,N,N*-trimethylglycine), which is used as an osmoprotectant by eukaryotes (including marine diatoms; Keller et al. 1999; Armbrust et al. 2004) and numerous bacteria (Imhoff and Rodriguez-Valera 1984; Landfald and Strøm 1986), which can accumulate intracellular concentrations of this compound on the order of 1 M. Glycine betaine can be produced from choline, a component of the common membrane lipid phosphotidylcholine (Sohlenkamp et al. 2000). The transport and conversion of choline to glycine betaine in bacteria is encoded by a widespread operon, *betTABI* (Bremer and Kramer 2000). In addition to osmoprotection, glycine betaine offers significant protection against cold stress in such diverse microorganisms as *Listeria monocytogenes* (Wemekamp-Kamphuis et al. 2004), *Yersinia enterocolitica* (Annamalai and Venkitanarayanan 2009), and *Bacillus subtilis* (Hoffmann and Bremer 2011). In each case, inactivation of osmoprotectant uptake transporters led to a decreased ability to withstand chill stress.

Although many bacteria are capable of importing and converting choline to glycine betaine for use as a compatible solute, few utilize it as an energy source. Glycine betaine degradation proceeds by serial demethylation to dimethylglycine, sarcosine, and glycine, which can be further catabolized to serine and pyruvate. Although the metabolic pathway is conserved, there are multiple non-homologous proteins capable of performing these reactions. *Pseudomonas aeruginosa* PAO1 produces two demethylases that generate dimethylglycine (GbcAB) and sarcosine (DgcAB; Wargo et al. 2008), while alternate genes have been reported from *Sinorhizobium meliloti* (Smith et al. 1988) and *Arthrobacter globiformis* (Meskys et al. 2001).

Sarcosine can be demethylated to glycine by a monomeric sarcosine oxidase (as in *Bacillus spp.*; Trickey et al. 1999), or by a heterotetrameric sarcosine oxidase (TSOX), as described in *Corynebacterium* (Chlumsky et al. 1995). The genes encoding TSOX in *Corynebacterium* are found closely packed with serine hydroxymethyltransferase (*glyA*) and formyltetrahydrofolate deformylase (*purU*) in the order *glyA*-*soxBDAG*-*purU*. In the reaction of TSOX, sarcosine is demethylated to glycine while O_2_ is reduced to H_2_O_2_ and formaldehyde is released, except in the presence of tetrahydrofolate, when 5,10-methylenetetrahydrofolate is released instead. The resulting compounds can be utilized by the cell for energetic or biosynthetic purposes: i.e. PurU catalyzes the recycling of 5,10-methylenetetrahydrofolate to tetrahydrofolate; GlyA interconverts glycine and l-serine. Energy in the form of reducing equivalents (i.e. NADH) can also be obtained by the conversion of formaldehyde to formate and then to CO_2_ by formaldehyde dehydrogenase (FdhGBAD) and formate dehydrogenase, respectively.

Here, we describe the horizontal transfer and partial duplication in *C. psychrerythraea* 34H of an operon responsible for the catabolism of sarcosine to l-serine. Genes enabling the importation and degradation of choline to glycine betaine and then sarcosine were also examined by similarity with experimentally verified genes in other microorganisms. A defined medium for the growth of *C. psychrerythraea* 34H, based on sarcosine, lactate, and vitamins (SLV), was developed on the basis of this genomic evidence. Numerous strains of *Colwellia* grew on this medium, indicating that compatible solute degradation is widespread in psychrophilic halophilic members of the genus *Colwellia* and may provide a selective advantage to these extremophiles in sea ice.

## Methods

### Phylogenomics

Microbial genomes encoding the glycine betaine catabolic pathway were investigated using tools available at the Joint Genome Institute Integrated Microbial Genomes (JGI-IMG) database v3.5 (http://img.jgi.doe.gov; Markowitz and Kyrpides 2007), containing 3183 complete or partial genomes for Bacteria and Archaea. Genomic islands were detected using IslandPath (Hsiao et al. 2003).

Small subunit 16S ribosomal RNA gene sequences from each bacterial genome in JGI-IMG were imported into ARB (Ludwig et al. 2004) from version 111 of the SILVA database of quality-checked, pre-aligned rRNA gene sequences. Additional bacterial reference sequences were added, including those from *Colwellia* species. 16S-rRNA gene sequences not yet available in SILVA were exported from JGI-IMG and aligned using the SINA Webaligner before being imported into ARB. For each subset of interest, highly variable base positions were trimmed from the nucleotide alignment (SILVA filter pos_var_ssuref:bacteria; mask ‘.’) and a maximum likelihood phylogenetic tree was inferred with PhyML v20120412 (options: model = TN93, base frequencies = ML, ts/tv = estimated, Pinv = estimated). To reduce computation time, several representative sequences were selected from the large monophyletic genera (*Pseudomonas*, *Shewanella*, and *Vibrio*).

The IMG Homolog Toolkit was used to identify homologs of *C.* *psychrerythraea* 34H sarcosine oxidase subunit alpha (SoxA). A total of 426 proteins were identified with bit scores greater than 400 and lengths of at least 900 amino acids. The amino acid sequences were exported from IMG and aligned using Clustal Omega v1.0.3 (options: –iter = 5). A maximum likelihood phylogenetic tree was inferred with PhyML v20120412 (options: −d aa) and imported into FigTree v1.3.1 for viewing. The multiple sequence alignment and phylogenetic inference were repeated for the Gammaproteobacterial subtree containing *C. psychrerythraea* 34H SoxA. The final tree included 47 Gammaproteobacterial SoxA sequences and 5 outgroup sequences. The genomic context of each homolog was examined at JGI-IMG; all 52 *soxA* homologs were in conserved gene arrangements of the form *soxBDAG*. Gene cassettes as determined by JGI-IMG are defined as consecutive ORFs separated by less than 300 bp each.

### Molecular evolution

To predict the time of introduction of the horizontally transferred sarcosine oxidase operon, calculations of reverse G + C content amelioration were performed using the techniques of Muto and Osawa (1987) and Lawrence and Ochman (1997), with the following changes. First, to avoid biases associated with non-independence of variables, GC3 was used as the independent variable rather than total G + C content. Second, non-linear functions relating GC3 to GC1 and GC2 were used to predict codon-position-specific G + C content. The functions were selected and their coefficients determined empirically using least-squares regression at the website http://www.zunzun.com, using a dataset of 3,980 bacterial and archaeal genomes and plasmids available from JGI-IMG. To obtain 90 % confidence intervals, 1,000 bootstrap replicates of each sequence (resampled by codon) were reverse-ameliorated for 1,000 time steps of 1 My each. Software to perform these calculations is freely available at http://www.github.com/rec3141/gcamel.

Divergence time estimates of paralogous genes were calculated using an empirical substitution rate of 0.91 % per synonymous site per My or 0.045 % per non-synonymous site per My (Lawrence and Ochman 1997). Synonymous and non-synonymous substitution rates were calculated by the method of Goldman and Yang (1994) using the program PAL2NAL (Suyama et al. 2006).

### Growth on compatible solutes

Defined medium containing sarcosine (*N*-methylglycine; 2 g L^−1^) was tested for its ability to support growth of *C.* *psychrerythraea* 34H as the sole source of organic carbon and nitrogen. *C.* *psychrerythraea* 34H was first grown at −1 °C from duplicate glycerol stocks in 50 ml of 1/2 × Marine Broth 2216. Growth was monitored by optical density at 600 nm; 15 mL aliquots were taken when an OD600 of 0.4 (>10^8^ cells mL^−1^) was reached. To rinse the inoculum of undefined medium, these aliquots were centrifuged at 1000×*g* for 10 min at 8 °C; the supernatant was discarded, 15 mL of ASW (NaCl 23.4 g L^−1^, MgSO_4_·7H_2_O 4.9 g L^−1^, CaCl_2_·2H_2_O 1.1 g L^−1^, KBr 0.2 g L^−1^, KCl 0.75 g L^−1^, MgCl_2_·6H_2_O 4.1 g L^−1^) was added without re-suspending the pellet and the tubes were centrifuged again at 1000×*g* for 2 min. The supernatant was again discarded, a final 15 mL of ASW was added and the pellet re-suspended. To three 50 mL aliquots of each experimental medium (and a positive control of 1/2 × Marine Broth 2216) was added 0.5 mL of rinsed inoculum, of which two were incubated at 4 °C and one at room temperature as a negative control.

Additional media were developed to increase the growth rate of *C.* *psychrerythraea* 34H on compatible solutes (choline, glycine betaine, and sarcosine) with the addition of lactate and vitamins (media denoted CLV, GBLV, and SLV, respectively). The growth of *C.* *psychrerythraea* 34H was tested on all three media; 9 additional *Colwellia* isolates were tested for growth on SLV. The recipe is as follows: choline *or* glycine betaine *or* sarcosine (2 g L^−1^) and calcium lactate (0.5 g L^−1^) are added to 1 L of TAPSO-buffered ASW and autoclaved. On cooling, 100 × RPMI-1640 vitamin solution (D-biotin 0.02 g L^−1^, choline chloride 0.3 g L^−1^, folic acid 0.1 g L^−1^, myo-inositol 3.5 g L^−1^, niacinamide 0.1 g L^−1^, p-amino benzoic acid 0.1 g L^−1^, d-pantothenic acid 0.025 g L^−1^, riboflavin 0.02 g L^−1^, thiamine·HCl 0.1 g L^−1^, vitamin B-12 0.0005 g L^−1^, KCl 0.2 g L^−1^, KH_2_PO_4_ 0.2 g L^−1^, NaCl 8 g L^−1^, Na_2_HPO_4_ 1.15 g L^−1^) is added to a final concentration of 0.5 mL L^−1^. To make solid medium, 4 g L^−1^ ultra-pure agarose may be added before autoclaving.

## Results and discussion

### Comparative genomics

#### Compatible solute catabolic pathway

**Fig. 1:**
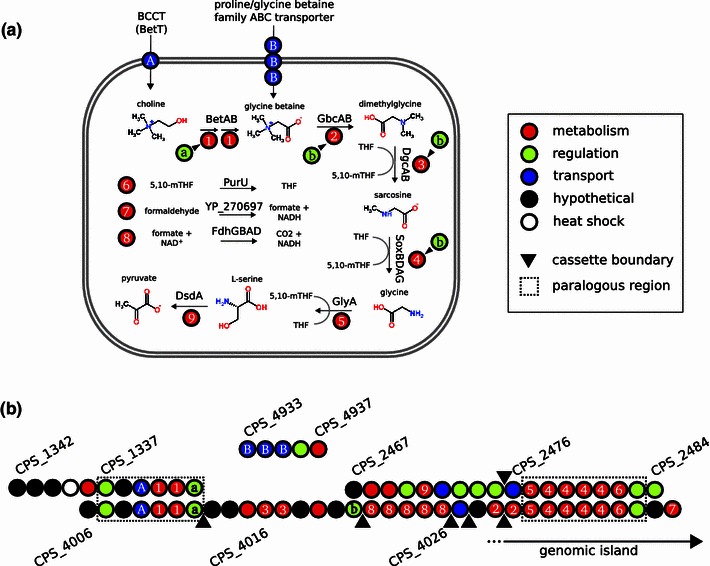
**a** The pathway of choline degradation to l-serine encoded by *Colwellia psychrerythraea* 34H. **b** Genes involved in the degradation process: *betAB* (CPS_4010–CPS_4011, CPS_1332–CPS_1333), *gbcAB* (CPS_4029–CPS_4030), *dgcAB* (CPS_4016–CPS_4017), *soxBDAG* (CPS_2478–CPS_2481, CPS_4032–CPS_4035), *glyA* (CPS_2477, CPS_4031, CPS_0728, CPS_3844), and *dsdA*, a truncated serine dehydratase (CPS_2471). Genes involved in one-carbon cyling and folate metabolism are: *purU* (CPS_2482, CPS_4036, CPS_4357, CPS_3620), *folD* (CPS_3133, CPS_3791), formaldehyde dehydrogenase (CPS_4039), and *fdhGBAD* (CPS_4022-4026, CPS_2056-2060). Genes for putative transporters for quarternary amines are: choline BCCT *betT* (CPS_4009, CPS_1335), putative sarcosine BCCT (CPS_3860), and a putative glycine betaine ABC transporter (CPS_4933–CPS_4935). Regulatory genes with predicted functions include: *betI* (CPS_4012 and CPS_1334; subscript ‘a’) and *gdbR* (CPS_4012; subscript ‘b’). *THF* tetrahydrofolate, *5,10-mTHF* 5,10-methylenetetrahydrofolate. *Dotted boxes* indicate regions of paralogy

The genome of *C.* *psychrerythraea* 34H contained genes encoding for importation and serial demethylation of choline to glycine betaine (*betTABI*; CPS_4009–CPS_4012, another copy was found at CPS_1332–CPS_1335), then to dimethylglycine (*gbcAB*; CPS_4029–CPS_4030), and finally to sarcosine (*soxBDAG*, CPS_4016–CPS_4017, another copy was found at CPS_2476–CPS_2473; Fig. 1). Directly flanking both sets of TSOX genes were homologs of *glyA* and *purU*, each of which was predicted to belong to the *soxBDAG* operon in the arrangement *glyA*-*soxBDAG*-*purU*. Both of these enzymes act directly on the products of TSOX: serine hydroxymethyltransferase (GlyA) catalyzes the reversible conversion of glycine to l-serine, while formyltetrahydrofolate deformylase (PurU) catalyzes the conversion of 5,10-methylenetetrahydrofolate to tetrahydrofolate and formate (Fig. 1). Genes encoding formaldehyde dehydrogenase (CPS_4039) and formate dehydrogenase (*fdhGBAD*; CPS_4022–CPS_4026, another copy was found at CPS_2056–CPS_2060) were also co-located on the chromosome, and perform further reactions on the products of glycine betaine degradation. Thus, the net reaction encoded by *C.* *psychrerythraea* 34H is the importation and complete degradation of choline, and potentially any intermediates, to l-serine (via glycine) and carbon dioxide (via formate).

#### Compatible solute transporters

Several transporters implicated in the importation of quaternary amine compounds were previously identified in the genome of *C.* *psychrerythraea* 34H (Methé et al. 2005), none of which were highly similar to previously characterized compatible solute transporters, such as ProU1 from *Vibrio parahaemolyticus*, OpuABC from *Pseudomonas aeruginosa* PAO1, or OpuAC from *Listeria monocytogenes*. Of the six predicted transporters, one was annotated as a primary transporter of the ATP-binding cassette (ABC) family and five were annotated as secondary transporters of the betaine–choline–carnitine transporter (BCCT) family. Two additional transporters were located in the vicinity of compatible solute degradation genes, but their substrates are as yet unknown: CPS_2472, an outer membrane porin, and CPS_2476, an antiporter of the NhaC family.

Although the putative proline/glycine betaine ABC transporter (CPS_4933–CPS_4935) was highly similar to a pair of ABC transporters from *P.* *aeruginosa* PAO1 (PA5096, PA5103), neither of these transporters were detected in this pseudomonad after induction by growth on glycine betaine (Diab et al. 2006) or by osmotic shock (Aspedon et al. 2006), nor were they required for growth on glycine betaine (Wargo et al. 2008). The substrates for these transporters are not yet known.

Transporters in the BCCT family have been shown to uptake at least 10 different compatible solutes to date (Ziegler et al. 2010). The five putative BCCT transporters found in the *C.* *psychrerythraea* 34H genome fell into three distinct classes based on sequence similarity. Two members of the *betT* class (CPS_1335 and CPS_4009) were 80 % identical at the amino acid level, located within *betTABI* operons, which convert choline to glycine betaine. These transporters are predicted to import choline. The second class included two genes, CPS_2003 and CPS_4027, which were 43 % identical at the amino acid level. Several homologs (52–57 % amino acid identity) in marine Gammaproteobacteria belonged to BCCT transporters implicated in the metabolism of dimethylsulphoniopropionate (DMSP; Todd et al. 2007; Johnston et al. 2008). In their respective genomes, these homologs are found as part of the *ddd* operon, which converts DMSP to dimethylsulfide (DMS), but in *C.* *psychrerythraea* 34H each of these genes stands alone. There are no other indications that *C.* *psychrerythraea* 34H metabolizes DMSP or DMS (e.g. it lacks the *ddd* operon), so the substrate for this class of BCCT transporters in *C.* *psychrerythraea* 34H remains speculative at this time. The final class of BCCT transporter in *C.* *psychrerythraea* 34H was represented only by CPS_3860. Homologs of this gene were found in other marine bacterial genomes, but no specific association with compatible solutes has been demonstrated.

#### Regulation of compatible solute metabolism

The expression of a niche-specific pathway, like compatible solute metabolism, in a generalist like *C.* *psychrerythraea* 34H can be expected to be well regulated. A number of putative regulators of choline and sarcosine metabolism were detected in the genome of *C.* *psychrerythraea* 34H, including a putative homolog of *gdbR* (CPS_4021; 59 % identity), an AraC-type transcriptional regulator known from *Pseudomonas aeruginosa* PAO1. This protein has been shown to control the expression of *gbcAB* and *dgcAB* (Wargo et al 2009). Immediately after each *soxBDAG* operon were predicted transcriptional regulators of the XRE family (CPS_2483 and CPS_4037) that could also be involved in differential regulation of sarcosine metabolic activity.

Another class of regulators, acting via cyclic diguanylate (c-di-GMP), have been shown to control motility, attachment, EPS production and biofilm formation in a number of Gammaproteobacteria, including various species of *Pseudomonas* (Gjermansen et al. 2006), *Vibrio* (Beyhan et al. 2008; Ferreira et al. 2008), and *Shewanella* (Thormann et al. 2006). *C.* *psychrerythraea* 34H encodes 65 regulatory elements associated with c-di-GMP—more than 90 % of all sequenced Gammaproteobacteria. The duplicated *glyA*-*soxBDAG*-*purU* operon may be in part regulated by a GGDEF domain protein (CPS_2484), based on the presence of the gene within the *glyA*-*soxBDAG*-*purU* gene cassette. Most of the known proteins containing GGDEF/EAL domains, including CPS_2484, contain transmembrane domains and signal reception and transduction domains with which they sense the external chemical environment.

### Phylogenomics

#### Phylogenetic distribution of glycine betaine catabolic genes

The use of glycine betaine as a compatible solute is widespread in Bacteria. More than 40 % of Gammaproteobacteria in JGI-IMG encode homologs of BetABI (responsible for the importation and conversion of choline to glycine betaine) including many members of the Orders Alteromonadales, Pseudomonadales, Oceanospirillales, and Vibrionales. In this study, we determined that the capacity to degrade this compatible solute is rarer—less than 25 % of glycine betaine utilizers also encode homologs of the catabolic pathway: glycine betaine demethylase (GbcAB), dimethylglycine demethylase (DgcAB), and sarcosine oxidase (SoxBDAG). Most of these glycine betaine degraders are Pseudomonadales and Oceanospirillales. We found 46 genomes of Gammaproteobacteria encoding SoxBDAG homologs: 34 members of the genus *Pseudomonas*; 10 marine halophiles of the Oceanospirillales; 1 *Vibrio*, and *C.* *psychrerythraea* 34H, an Alteromonadales.

Several lines of evidence indicated that *soxBDAG* and other genes in the *C.* *psychrerythraea* 34H genome were horizontally transferred, probably from marine Gammaproteobacteria of the Order Oceanospirillales. The complete absence of the glycine betaine degradation pathway in existing genomes of most genera belonging to the Alteromonadales suggests that the genes were not simply retained in *C.* *psychrerythraea* 34H while being lost in all of the rest. Multiprotein phylogenetic trees consistently show an ancient divergence between one group comprised of Pseudomonadales and Oceanospirillales and a second group of Vibrionales, Alteromonadales, Aeromonadales, and Pastuerellales (Williams et al. 2010). We suggest that glycine betaine degradation is an ancient trait of the Pseudomonadales/Oceanospirillales clade, which has been horizontally transferred into the genomes of (at least) two members of the Vibrionales/Alteromonadales/Aeromonadales/Pastuerellales group since the divergence. The mechanism of horizontal gene transfer by which *C.* *psychrerythraea* 34H acquired its exogenous genes was not determined in this study, but the likelihood that *C.* *psychrerythraea* 34H is a lysogen has been explored by Wells (2006), suggesting transduction as a possible mechanism.

**Fig. 2:**
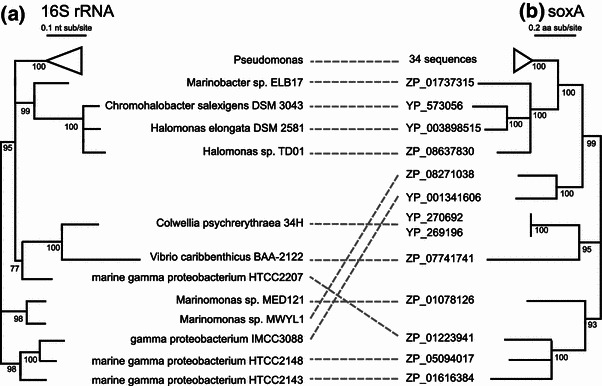
**a** Maximum likelihood phylogenetic tree of 16S rRNA gene sequences from gammaproteobacteria genomes in the JGI-IMG database containing SoxA homologs. **b** Maximum likelihood phylogenetic tree of amino acid sequences encoding heterotetrameric sarcosine oxidase subunit alpha (SoxA) present in the JGI-IMG database. *Node labels* indicate approximate likelihood ratio test (aLRT) support values as percentages; nodes with aLRT support values less than 70 % were collapsed into hard polytomies. *Dashed lines* match the 16S rRNA and SoxA sequences from the same organism; *crossed lines* indicate topographic mismatches, possibly due to horizontal gene transfer

Phylogenetic analysis of SoxA homologs demonstrated that the nearest sequenced homolog of *C.* *psychrerythraea* 34H SoxA was in *Vibrio carribbenthicus*, though the complete absence of SoxA in dozens of other *Vibrio* genomes suggests that this homolog was also acquired by horizontal gene transfer (Fig. 2). Poor phylogenetic resolution at interior nodes of the SoxA tree make deeper relationships murky. The most taxonomically similar genome to contain *soxBDAG* was *Marinobacter* sp. ELB17, formally described as Order Alteromonadales but phylogenetically more similar to Order Oceanospirillales (Fig. 2 and Williams et al. 2010). The remainder of the SoxA homologs belonged to members of the Pseudomonadales and Oceanospirillales.

#### Evolutionary history of compatible solute utilization in *C.* *psychrerythraea* 34H

The acquisition of compatible solute utilization genes in *C.* *psychrerythraea* 34H appears to have been a stepwise process occurring over the course of the last several hundred million years of Earth history. Although much of the Phanerozoic Eon (541 Mya to today) was warmer than the present day and lacked polar ice caps, the inferred timing of significant events in the evolution of *C.* *psychrerythraea* 34H reflect geological periods when polar ice existed.

Two copies of the *betTABI* operon were located on the *C.* *psychrerythraea* 34H chromosome. The gene clusters (CPS_1337–CPS_1332 and CPS_4007–CPS_4012) appeared as ancient paralogs in phylogenetic analyses (not shown). Divergence time estimates of individual genes in the cluster ranged from 236 to 1879 Mya, with a median age of 298 My (Table 1). This timing coincides with that of the Karoo glaciation, which had a maximal extent around 300 My ago (Crowley and Baum 1992).

**Table 1:** Divergence rates and times of paralogous gene clusters involved in compatible solute utilization in *C.* *psychrerythraea* 34H

Gene	Locus 1	Locus 2	S	N	dS	dN	dN/dS	*t*_*S*_	*t*_*N*_
MarR regulator	CPS_4007	CPS_1337	110	335	2.68	0.27	0.10	294	605
Hypothetical	CPS_4008	CPS_1336	159	540	2.58	0.26	0.10	283	572
*betT*	CPS_4009	CPS_1335	328	887	3.72	0.13	0.03	409	281
*betA*	CPS_4010	CPS_1334	385	1295	2.18	0.11	0.05	239	236
*betB*	CPS_4011	CPS_1333	349	1112	17.09	0.11	0.01	1879	241
*betI*	CPS_4012	CPS_1332	139	449	2.75	0.26	0.09	302	567
*glyA*	CPS_4031	CPS_2477	313	938	0.18	0.00	0.02	19	7
*soxB*	CPS_4032	CPS_2478	249	999	0.00	0.00	0.00	0	0
*soxD*	CPS_4033	CPS_2479	49	248	0.00	0.00	0.00	0	0
*soxA*	CPS_4034	CPS_2480	675	2346	0.00	0.00	0.00	0	0
*soxG*	CPS_4035	CPS_2481	213	381	0.01	0.00	0.00	1	0
*purU*	CPS_4036	CPS_2482	210	666	0.08	0.00	0.00	9	0.2
DNA binding protein	CPS_4037	CPS_2483	155	439	0.36	0.04	0.10	40	80

*S* number of non-synonymous sites, *N* number of synonymous sites, *dS* synonymous substitution rate, *dN* non-synonymous substitution rate, *t*_*S*_ synonymous site divergence (Myr), *t*_*N*_ non-synonymous site divergence (Myr)

A 37 kbp genomic island (base pairs 4,236,340–4,273,184; loci CPS_4030–CPS_4060) was identified based on abnormal dinucleotide bias, and G + C content (45–49 %) exceeding 2 standard deviations from the mean genomic G + C content (38.0 ± 3.5 %). The genomic island contained the *glyA*-*soxBDAG*-*purU* operon and other genes involved in 1-carbon metabolism. An analysis of the amelioration of the G + C content of the 2435 codons encoding the *glyA*-*soxBDAG*-*purU* operon predicted that the G + C content of the donor microorganism was about 55 % (90 % CI 53–57 %), similar to that of *Marinobacter* sp. ELB17 (54 %). In addition, the G + C amelioration analysis inferred an estimated time since introduction of 150 My (90 % CI: 70–220 My). This timing approximates a period of cooling in the Late-Jurassic/Early-Cretaceous (about 160 Mya) that may have precipitated a brief ice age in an otherwise warm era (Dromart et al. 2003).

Two copies of the *glyA*-*soxBDAG*-*purU* operon were located on the *C.* *psychrerythraea* 34H chromosome. The first set was located within the previously described genomic island; the second set was duplicated from the first in the more recent past, along with a regulatory gene encoding a DNA-binding protein. Of the 70 nucleotide substitutions observed in the 7561 bp region encoding *glyA*-*soxBDAG*-*purU*, most occurred at the edges of the operon, within *glyA* and *purU*. Only two substitutions were observed throughout *soxBDAG*, both synonymous substitutions in *soxG*. The DNA-binding protein exhibited a greater substitution rate (56 synonymous and 16 non-synonymous changes). Based on the observed substitution rates, this duplication event likely occurred within the last few millions years (Table 1) and thus since the re-establishment of polar ice caps in the Miocene (Zachos et al. 2001).

Any method of molecular clock dating is subject to error and the ages described here are no exception. However, the order of the described events is much more reliable, is internally consistent, and is logical. The reconstructed order of events is as follows: originally, *C.* *psychrerythraea* 34H, like most extant Alteromonadales, utilized glycine betaine as an osmoprotectant but was not able to degrade it. At some point, the *betTABI* operon was duplicated, perhaps allowing increased or fine-tuned expression of glycine betaine use, or neofunctionalization to allow the importation of related compounds. Next, the genomic island encoding sarcosine oxidase was horizontally transferred into the genome. We did not attempt to date the origin of the glycine betaine demethylase (GbcAB) or dimethylglycine demethylase (DgcAB), but their co-location on the genome suggests they may have arrived coincident with the genomic island. At this point, *C.* *psychrerythraea* 34H would have gained the ability to degrade glycine betaine. Finally, much later, the sarcosine oxidase operon was duplicated; it has not yet had time to functionally diverge. A future line of research will be to determine the role these paralogs play in the adaptation of *C.* *psychrerythraea* 34H to cold, salty environments.

### Phenotypes

#### A defined medium for *C.* *psychrerythraea* 34H

Perhaps the most important qualification to define a successful horizontal gene transfer event is endogenous expression of the transferred genes in the recipient. Sarcosine was tested as a sole source of organic carbon and nitrogen for *C.* *psychrerythraea* 34H growth. Turbid growth (*OD*600 > 0.1; > 10^7^ cells mL^−1^) was observed in duplicate aliquots of the sarcosine medium after 5 weeks. Successful growth after two consecutive transfers in the sarcosine medium demonstrated that growth was not dependent on co-factors remaining from prior growth in undefined medium. In an effort to increase the growth rate of *C.* *psychrerythraea* 34H on sarcosine, we amended the medium with calcium lactate, vitamin solution, or a combination of both. We observed growth on the combined medium, denoted SLV (for sarcosine, lactate, and vitamins) after 1 week of incubation at 4 °C. After 5 weeks, no growth of *C.* *psychrerythraea* 34H was detected in ASW containing only lactate or only vitamin solution without sarcosine. When compared with growth on 1/2 × Marine Broth 2216 at the same temperatures, the time required for growth of *C.* *psychrerythraea* 34H in SLV was slower (by about a factor of 2) and to a lower *OD*600 (<0.2 in SLV as compared to >0.4 in rich media). Defined media containing lactate, vitamins, and either choline (CLV) or glycine betaine (GBLV) were also tested for their ability to support growth of *C.* *psychrerythraea* 34H; growth to turbidity was observed in each medium within 10 days at 4 °C.

#### Degradation of compatible solutes by *Colwellia* spp.

**Fig. 3:**
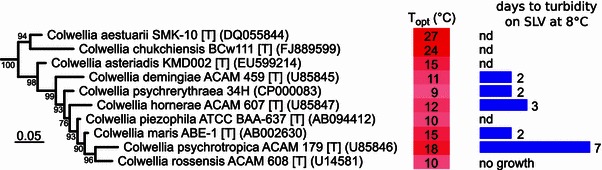
Maximum likelihood phylogenetic tree of 16S rRNA gene sequences from *Colwellia* isolates. *Node labels* indicate approximate likelihood ratio test (aLRT) support values as percentages. *Background shading* indicates optimum growth temperature (°C) for each strain. Growth on defined medium SLV at 8 °C is indicated as ‘days to turbidity’; *nd* not determined

To determine the taxonomic range of growth on sarcosine among *Colwellia spp.*, we tested the ability of 9 isolates to grow in liquid SLV at 8 °C. Growth on SLV was widespread throughout *Colwellia* (Fig. 3). All, but one (*C. rossensis* ACAM 608) grew on SLV and became turbid within 1 week. Three strains had no available sequence data but were phenotypically identified as *Colwellia spp.*: *C.* *demingiae* strain ICP10 (3 days to turbidity; J. Bowman, UT), Arctic sea ice isolate 21C (3 days to turbidity; Borriss et al. 2003), and Arctic nepheloid layer isolate 75C3 (7 days to turbidity; Wells 2006). Four *Colwellia* strains tested for growth on SLV were originally isolated from Antarctic sea ice diatom assemblages (Bowman et al. 1998). Three of these strains grew on SLV: *C.* *demingiae* ACAM 459T, *C.* *psychrotropica* ACAM 179T, and *C.* *hornerae* ACAM 607T. Sources of compatible solutes in sea ice are likely plentiful, including compounds released by diatoms and the lysis of bacteria by phage, which are well-known from sea ice (Maranger et al. 1994; Wells and Deming 2006a). The ability of *Colwellia spp.* to grow on compatible solutes as a sole source of organic carbon and nitrogen suggests the possibility of bringing into culture as-yet-uncultured microorganisms from environments expected to contain high levels of compatible solutes, including sea ice and other frozen saline environments.

## Conclusions

In a previous analysis of the genome of *C.* *psychrerythraea* 34H, a psychrophilic, halophilic marine bacterium, Methé et al. (2005) identified a duplicated operon encoding for heterotetrameric sarcosine oxidase (SoxBDAG), an enzyme involved in the catabolism of glycine betaine, a common osmoprotectant molecule. In the present genomic analysis we have identified this operon as the result of a horizontal gene transfer event. Molecular evolutionary estimates of important events in the adaptation of *C.* *psychrerythraea* 34H to compatible solute utilization appear to recapitulate the geological evolution of the polar regions. We have also demonstrated the genetic potential for *C.* *psychrerythraea* 34H to use both choline and glycine betaine as substrates for growth. Furthermore, we successfully grew *C.* *psychrerythraea* 34H on a medium containing sarcosine as the sole source of carbon and energy, which was then developed into SLV, a defined medium for the rapid growth of many *Colwellia* species. With these developments, *Colwellia* species, which previously could only be grown rapidly in Marine Broth, can be included in more defined and sophisticated studies of its strategies to survive and compete in extreme environments.

## Ethics

Experiments complied with the current laws of the United States of America. The authors declare that they have no conflict of interest.
